# Stakeholders’ perception on including broader economic impact of vaccines in economic evaluations in low and middle income countries: a mixed methods study

**DOI:** 10.1186/s12889-015-1638-0

**Published:** 2015-04-10

**Authors:** Ingeborg M van der Putten, Silvia MAA Evers, Rohan Deogaonkar, Mark Jit, Raymond CW Hutubessy

**Affiliations:** CAPHRI, School of Public Health and Primary Care, Department of Health Services Research, Maastricht University, Maastricht, The Netherlands; Initiative for Vaccine Research, World Health Organization, 20 Avenue Appia, 1211, Geneva 27, Geneva, Switzerland; Health Economics Unit, University of Birmingham, Birmingham, UK; Department of Infectious Disease Epidemiology, London School of Hygiene and Tropical Medicine, London, UK

**Keywords:** Mixed methods, Economic evaluations, Externalities, Vaccines, Immunization, Decision making, Low and middle income countries, Developing countries

## Abstract

**Background:**

Current health economic evaluation guidelines mainly concentrate on immediate health gains and cost savings for the individual involved in the intervention. However, it has been argued that these guidelines are too narrow to capture the full impact of vaccination in low and middle income countries. The inclusion of broader economic impact of vaccines (BEIV) has therefore been proposed. Some examples of these are productivity-related gains, macro-economic impact, and different externalities. Despite their potency, the extent to which such benefits can and should be incorporated into economic evaluations of vaccination is still unclear. This mixed methods study aims to assess the relevance of BEIV to different stakeholders involved in the vaccine introduction decision making process.

**Methods:**

In this mixed method study an internet based survey was sent to attendees of the New and Underutilized Vaccines Initiative meeting in Montreux, Switzerland in 2011. Additionally, semi-structured interviews of 15 minutes each were conducted during the meeting. Study participants included decision makers, experts and funders of vaccines and immunization programs in low and middle income countries. Descriptive analysis of the survey, along with identification of common themes and factors extracted from the interviews and open survey questions was undertaken.

**Results:**

Evidence on macro-economic impact, burden of disease and ecological effects were perceived as being most valuable towards aiding decision making for vaccine introduction by the 26 survey respondents. The 14 interviewees highlighted the importance of burden of disease and different types of indirect effects. Furthermore, some new interpretations of BEIVs were discussed, such as the potential negative impact of wastage during immunization programs and the idea of using vaccines as a platform for delivering other types of health interventions. Interviewees also highlighted the importance of using a broader perspective in connection to measuring economic impacts, particularly when attempting to derive the value of newer, more expensive vaccines.

**Conclusion:**

According to participants, BEIVs were seen as being equally important as traditional outcome measures used in cost-effectiveness analyses. Such insight can be used to shape research agendas within this field and to eventually create broader, more inclusive practical guidelines for economic evaluations of vaccines.

**Electronic supplementary material:**

The online version of this article (doi:10.1186/s12889-015-1638-0) contains supplementary material, which is available to authorized users.

## Background

It is estimated that in 2013, 6.3 million children worldwide died before turning five, equal to almost 17.000 children every day [1]. The leading causes of under-five mortality among children have been found to be pneumonia, other acute respiratory infections, and childhood diarrhea [1,2]. Despite a commendable average reduction of 49% from 1990 to 2013 [1], the decrease in current mortality rate will need to be quadrupled in order to reach the Millennium Development Goal of a two-thirds reduction in child mortality by 2015 [2]. It is estimated that an extra 4.2 million lives could be saved by ensuring access to comprehensive vaccine coverage [3]. However, low and middle income countries (LMICs) and international donors alike only have limited resources at their disposal. Hence, governments and donors have to trade off purchasing vaccines against other health care investment decisions [4,5]. Economic tools such as cost-benefit (CBA) and cost-effectiveness analysis (CEA) have the potential to strengthen the use of evidence in such a decision making context [6], hence creating a more transparent framework for vaccine introduction. Vaccine introduction can be interpreted as the addition of a vaccine to the Expanded Program on Immunization (EPI program), the introduction of a new product formulation that was already part of the program, a new combination vaccine, or a new route of administration for an already covered vaccine [7].

One of the key issues during vaccine introduction, besides issues related to the disease and the strength of the immunization program and the health system, is the vaccine itself in terms of safety, efficacy and the economic and financial consequences of introducing the vaccine [7]. Several guidelines on economic evaluations of vaccines have been published by research groups and international organizations to strengthen evidence based decision making during vaccine introduction [8-12]. The core components of these guidelines revolve around estimating the costs and benefits of vaccines and vaccination programs. Benefit components traditionally include health care cost savings or improvements in life expectancy and quality of life. Indirect benefits realized by caregivers and the greater community and/or effectiveness of the vaccine, as part of a larger program, are generally not included in these guidelines [13].

A report developed for the World Bank’s Human Development Network (Health, Nutrition and Population Family series) suggests that policy makers do not base their decision solely on the CEA results currently prescribed due to a lack of transparency and inability to factor in country specific contextual factors [4]. Moreover, it has been argued that the use of CEA alone may be too narrow to capture the overall impact of vaccination [14-18] and other child health programs [19]. In line with this report several initiatives, such as the Pan American Health Organization’s (PAHO) ProVac Initiative in Latin America [6,20], strengthen the process of national decision making by providing better infrastructure and tools for economic analysis, including training, data collection and analysis and general advocacy for the use of economic evidence in decision making [6]. Their program ProVac trains national teams to develop policy briefs using a framework with technical, financial, operational and social criteria.

Recent reviews of the broader economic impact of vaccination (BEIVs) have identified several domains that are not adequately captured by traditional metrics such as morbidity, mortality and generic health utility measures such as Disability Adjusted Life Years (DALY) and Quality Adjusted Life years (QALY) (see Table 1) [4,14,16,19,21-28]. However, some of the broader domains discussed (e.g. behavior-related productivity gains), are based on theoretical concepts and have not been subject to adequate empirical quantification. It is therefore important to assess the relevance of including some of these broader economic considerations in economic evaluation studies for various stakeholders, prior to their inclusion in research and practical guidelines. Hence, the goal of this study is to gauge the importance of the various BEIVs from a stakeholder perspective, and clarify the extent to which BEIVs can and should be incorporated into the guidelines for economic evaluations of vaccines to support policymakers and external funders in making decisions on vaccine introduction.

**Table 1:** **Categorized list of the economic impact of vaccination**

**Category**	**Definition**
*A. Burden of disease*	
1. Morbidity	Cases averted [14]
2. Mortality	Deaths averted [14]
3.Quality of life measures	DALYs and QALYs [14,16]
4. Health care cost savings	Reduction in cost of health care borne by the public sector or private individuals [16,23]
5. Governmental savings	Reduction in overall costs of government expenses. [21]
*B. Productivity-related gains* [14,22]	
1. Care-related productivity gains	Reduction in lost days of work due to sickness or caring for a sick patient
2. Outcome-related productivity gains	Increased lifetime productivity due to better health improves cognition, educational attainment and physical strength
3. Behavior-related productivity gains	Economic improvements due to changes in household choices such as fertility and consumption/saving as a result of improved child health and survival
*C. Ecological effects*	
1. Prevalence of drug resistance	Vaccination can prevent disease and thus obviate the need for antibiotic use, reducing the prevalence of antibiotic-resistant strains [14,22]
2. Serotype replacement effects	After the introduction of vaccine, non-vaccine serotypes may well replace vaccine serotypes, leading to a smaller reduction in disease burden over time [24].
3. Herd effect	Benefits accruing because vaccination improves outcomes among unvaccinated community members [21]
*D. Indirect effects*	
1.Equity	More equal distribution of health outcomes [4,19].
2. Interaction with other interventions	Events happening during the evaluation period not related to the intervention [4,25].
3. Health resources	Impact of vaccine programs on amount of health resources available (time, availability) [25]
4. Priority of interventions	Overlooking importance of social determinants of health by focusing on ‘silver bullets’ and ‘mass campaigns’ instead of adapting interventions to the prevailing culture and socioeconomic conditions, which generate the felt needs. [23]
*E. Macro-economic impact*	
1. Burden on other sectors	Macro-economic effect of vaccines on other sectors during epidemics [26]
2. School absenteeism	Amount of schooldays missed due to illness [16,27,28].

## Methods

Our study utilized a mixed method triangulation convergence model design [29]. This design allowed for simultaneous collection of both quantitative and qualitative data but also permitted separate analysis of the two components prior to comparing their respective results. The rationale for choosing this approach was to allow our qualitative findings to elucidate the quantitative data [29]. The qualitative component consisted of face-to-face interviews (further referred to as interviews with interviewees) and the quantitative component involved the use of an internet based survey (further referred to as survey with respondents). Both interviews and survey were concurrently conducted among the participants of the New and Underutilized Vaccines Initiative (NUVI) meeting in Montreux (Switzerland) in May 2011. This meeting was selected because of good representation from all major stakeholders involved in vaccine introduction. A wide range of stakeholder groups were represented including donor programs such as Global Alliance for Vaccines and Immunization (GAVI alliance) and the Bill and Melinda Gates Foundation, international organizations such as United Nations International Children’s Emergency Fund (UNICEF) and World Health Organization (WHO), pharmaceutical companies, research institutes, national managers for the EPI program, and other government or non-government (PATH, AMP, etc.) representatives.

### Ethical clearance

According to the Maastricht Ethical Review Committee (METC) “no ethical approval was required as the study is not concerned with medical research”.

### Quantitative data collection and analysis

An email was sent to the respondents one week prior to the NUVI meeting inviting them to participate in an online survey consisting of three parts: (i) introductory questions about the professional background of the respondents and expertise (ii) questions using a 5-point Likert-scale on the importance of different organizations involved in the decision making process, types of evidence used and scenarios gauging the importance of availability of BEIVs for decision making, (iii) three open questions about the importance of different types of evidence currently used for decision making on vaccine introduction. The different scenarios were based on various types of effects and outcomes documented in the literature (see Table 1) [4,14,16,19,21-28]. These types were categorized into five domains, i.e. burden of disease, productivity-related gains, ecological effects, indirect effects and macro-economic impact (Additional file 1).

First, among all respondents, a median was calculated for every identified impact separately. Second, medians were calculated for every domain by first calculating the means for every respondent separately per domain. These means were used to calculate the median per domain among all respondents. Medians of 5 were regarded as very important, 4 as important, 3 as more or less important, 2 as somewhat important and 1 as not important. The calculated medians per domain were visualized by using box plots to give insight in their relative importance. Third, subgroup analysis was conducted to compare medians per domain from different geographical working areas (global, low income, middle income) and by institutional affiliation (government, international organization, research institution). The geographical working areas were based on the World Bank GDP classification of countries in 2011 [30].

### Qualitative data collection and analysis

Respondents of the quantitative survey were offered the opportunity to self-select themselves into the interview sample. Interviewees were also personally recruited by RH, IvdP, MPVA and MJ during meeting breaks. The interview questions were exploratory in nature and based on themes covered in the survey. Interviewees were first asked to describe the current decision making process in the country(ies) or region(s) they were responsible for. Subsequent questions covered the applicability of BEIVs to their jurisdiction; any known economic evaluation cases wherein evidence alluding to a BEIV has been included and the methodology utilized for its measurement; the relevance of BEIVs when applied to both traditional EPI vaccines and newer, next generation vaccines (Additional file 2). Representatives from all relevant stakeholder groups were interviewed by either one or two interviewers.

The interview transcripts, interviewer notes and answers to the open-ended survey questions were summarized and analyzed by IP using a qualitative data analysis program (Nvivo). This was done by extracting basic themes from each of the different sources, using the theoretical framework underlying the survey as a reference point. Per effect, the specific information mentioned in the interviews was listed and quotes were selected. New topics were added into the framework if one interviewee mentioned a topic that was not previously captured. All authors discussed accuracy of the analyses and consensus was obtained in case of disagreement.

### Merging of separate analyses

Survey responses and interview comments were combined for each domain. Interviewee comments were used to explain results or to elucidate outcomes by giving practical examples of specific BEIVs.

## Results

**Figure 1:**
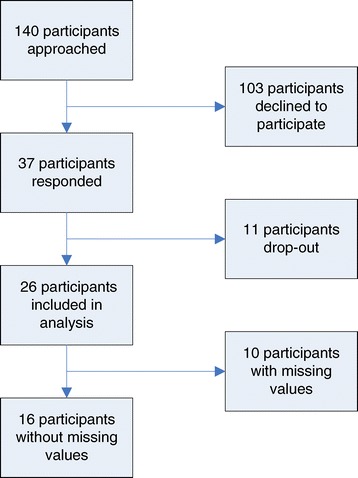
Flowchart respondents of survey.

Out of 140 respondents invited, 26 completed the survey in full while 11 respondents commenced but did not complete any of the analyzed open or Likert-scale questions. Of the 26 surveys included in the final analysis, 10 contained at least one or more questions with a missing response (see Figure 1). Survey respondents were of an average age of 45 years (SD 8.34), with an equal gender split. The average work experience of respondents in the field of immunization was 14.6 years (SD 10.75). Four worked exclusively in low income countries (LICs) and seven in middle income countries (MIC). Fifteen respondents worked in a mixed LMICs environment. In total, all respondents belonged to one of five stakeholder groups being represented at the meeting (see Table 2).

**Table 2:** **Distribution of institutional background (N = 26)**

**Institutional background**	**N = 26**	**Percentage (%)**
Government	4	15.4%
International organization	12	46.2%
Research institute	5	19.2%
Donor	3	11.5%
Vaccine manufacturer	2	7.7%

Seven interviewees volunteered to be interviewed for the qualitative portion of our study. A further 7 attendees agreed to participate after being approached during the NUVI meeting. Members of all stakeholder groups were interviewed (see Table 3). All continents, except Australia, were represented in the qualitative interviews. Specifically, two interviewees worked exclusively with LICs, six with MICs and six with LMICs.

**Table 3:** **Background characteristics of interviewees**

**Gender**	**Continent**	**Area of core interest**	**Stakeholder group**
M*	Africa	Low income country	International organization
F	South America	Middle income country	Vaccine manufacturer
M	Europe	Global	International organization
M	Europe	Middle income country	Government
M*	Europe	Global	Research institute
M*	North America	Global	International organization
M*	South America	Middle income country	Research institute
F*	Africa	Global	Donor
M	Asia	Middle income country	Government
F	Asia	Global	Research institute
M	Asia	Global	Donor
M*	South America	Middle income country	International organization
M	Asia	Low income country	Government
F*	South America	Middle income country	Government

*Interviewee completed the survey and volunteered to be interviewed.

### Current decision-making process

Survey respondents identified the Ministry of Health, Ministry of Finance and special expert advisory groups, such as National Immunization Technical Advisory Groups (NITAGs - named by thirteen respondents) [31] and Inter-agency Coordinating Committees (ICCs - named by four respondents), as being most important during the decision making process around new vaccines. One interviewee also underlined the increased role of NITAGs in decision making by providing government with recommendations based on all available vaccine specific evidence. The decision-making process has thus become more formal and scientific than before due to the inclusion of NITAGs. Survey respondents also cited the importance of local advisory groups (mostly professional associations). The parliament, international organizations such as Medicines Sans Frontier (MSF), UNICEF and WHO were seen as more or less important (median of 3).

Survey respondents stated that effectiveness data, burden of disease, cost-effectiveness data, overall costs of immunization program, public sector budget impact and evidence of vaccine safety were all seen as being very important considerations for making a well-balanced decision. Accessibility, applicability, credibility, the availability of other interventions, and equity/fairness were also worth considering (medians between 3 and 4) but in the context of decision making were viewed as being not as important as the other types of evidence above.

Interviewees stated that the decision-making process around vaccine implementation in LMICs is quite diverse per country. According to a non-governmental organization (NGO) representative working in Africa, most African countries use data on mortality rates, cases averted and absenteeism as the core for decision making. Absenteeism numbers are collected together with evidence on vaccine coverage in Brazil as well. No evidence on QALYs or DALYs as an isolated measure is believed to be used for decision making in most LMICs. In one Asian LIC, evidence on disease burden (mortality, morbidity and DALYs) and health sector impact are used to convince the Minister of Finance to invest in new vaccines. Next to disease burden, impact on productivity was observed by looking at school attendance or working days lost. However, no set decision threshold is used. Vaccine costs and safety also play a role. For example, the Human Papillomavirus (HPV) vaccine targets an area of high disease burden but is also very costly and is therefore not implemented. Lastly, the importance of “herd effects” was acknowledged but no explicit mechanisms for their inclusion were declared.

### Relative importance of domains

**Figure 2:**
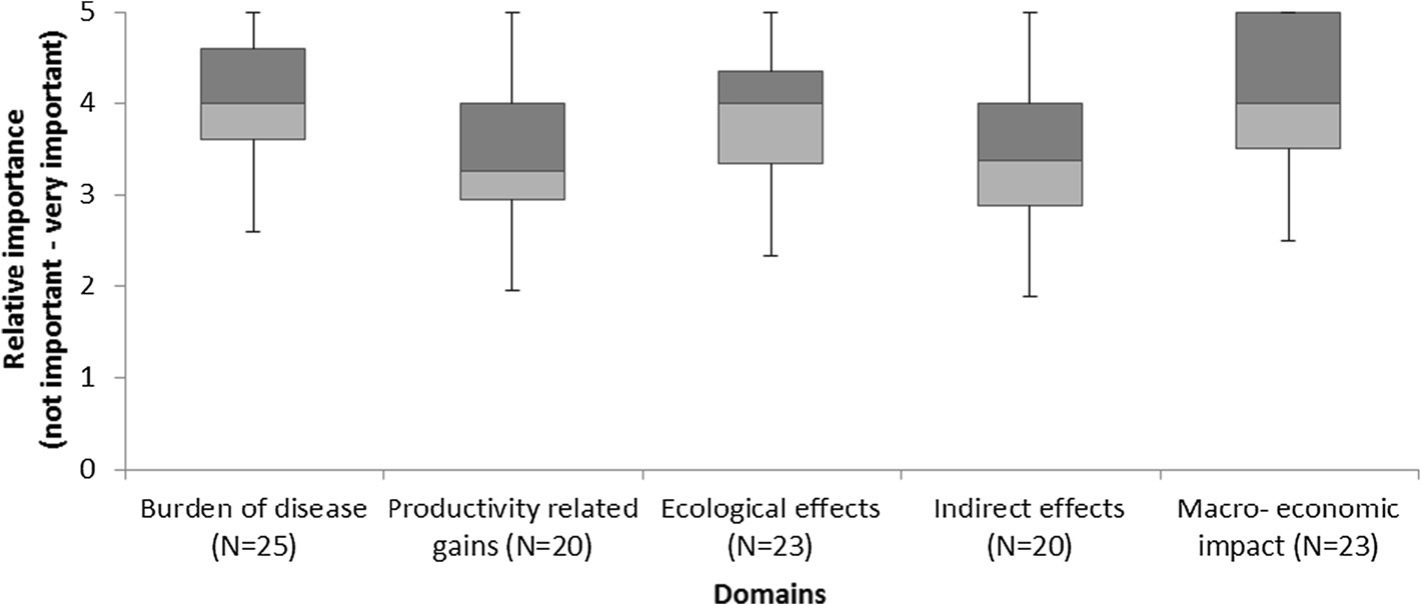
Boxplots importance of providing evidence on vaccine related issues per domain.

Macro-economic impact, burden of disease and ecological effects were observed to be the most valuable domains according to the quantitative survey (Figure 2). All scored above four on the Likert-scale, which indicates very important. Specifically, evidence on herd effects was viewed as most important, closely followed by governmental cost savings, deaths averted and burden on other sectors. Productivity-related gains and indirect effects were seen to be somewhat valuable. The relatively low Likert-scores for productivity-related gains can be partly attributed to the low score for productivity outcomes relating to household behavior. For the indirect effects domain, the effects measured were all observed to be somewhat valuable. Detailed results providing an account of the importance of specific effects within each domain follow.

#### Burden of disease

Overall survey responses and four interviewees asserted the importance of burden of disease as being the most powerful evidence type in support of vaccine implementation. Two interviewees regarded burden of disease to be the primary basis for implementation with other evidence types being included for purposes of balanced appraisals. Another interviewee expressed that outcomes demonstrating a reduction in burden of disease, such as reduction in under-five mortality, as compelling evidence capable of attracting further investment by potential funders outside the health sector. Similarly, evidence on deaths averted was viewed as being more important than the amount of cases averted. The fourth interviewee highlighted the general importance of burden of disease but did not elaborate on its higher relevance. Three interviewees mentioned the importance of morbidity together with mortality as they are often used in CEA studies. However, one interviewee did not consider morbidity to be a very important outcome as it “only reflects reduction in contraction of the disease” (sic).

Survey respondents generally ranked QALYs as being very important. During the interviews most of the interviewees talked about QALYs or DALYs interchangeably. Interviewees mentioned that the use of QALYs or DALYs depends on the country specific availability of such type of indicators. Measuring them was seen as valuable, but capacity building of local expertise to measure and value quality of life indicators was also needed. One interviewee also mentioned the complicated nature of using QALYs and DALYs in decision making, as the outcomes are difficult to explain to decision makers not trained in economics and public health.

Providing evidence on the impact of vaccines on health care costs was seen as being just as important as evidence on morbidity. One participant asserted that a reduction in disease-related health expenditures could be a possible effect of vaccine implementation. Two interviewees considered budgetary impact to be one of the main ways of convincing decision makers to invest in immunization programs, while a third interviewee saw evidence of vaccine impact on the health care budget as an aim for conducting CEA research. To underline its importance, one of the interviewees mentioned that budget impact analysis was considered to be the most important type of economic evidence in MICs such as India and Indonesia.

Governmental cost savings was another factor. The impact of a vaccine on the national budget was mentioned by one of the survey respondents. Only one interviewee discussed the importance of impact on the entire governmental budget. He regarded it to be valuable evidence as ultimately the department for development and planning and Minister of Finance makes the final decision.

#### Productivity-related gains

Seven out of fourteen interviewees alluded towards the possibilities of measuring productivity gains. Only one interviewee said something about all three types of effects included within this domain, while the others only elaborated on outcome-related productivity gains.

Compared with the other types of productivity gains mentioned in the survey, outcome-related productivity gains were viewed as being most relevant. One interviewee reflected on how a polio outbreak in his country also led to a loss of production over time due to the cognitive impact of the disease on patients. Importantly, interviewees were aware of some issues limiting the relevance of outcome-related productivity gains. Firstly, one interviewee recognized that despite its theoretical validity, the argument that child immunization could lead to increased productivity later in life was hardly ever brought up during discussions with Ministries of Health. Secondly, two other interviewees acknowledged the added value of such evidence but also pointed to difficulties in measuring such an effect due to insufficient data. Thirdly, another interviewee only partly agreed on the applicability of outcome-related productivity gains as they felt that it could only be used to evaluate benefits of certain vaccines targeting diseases, such as Japanese Encephalitis, which leads to some sort of cognitive damage in a third of the infected children. A further three interviewees were less convinced about the applicability of outcome-related productivity gains, stating that these gains were already implicitly included in the calculation of benefits and that it only provides a parental perspective rather than a governmental one.

Care related productivity gains were acknowledged by 3 interviewees and further discussed by one of them. This interviewee mentioned that care-related productivity gains could be more relevant in the context of high income countries (HICs) rather than LMICs. For example, the introduction of the chicken pox (varicella) vaccine in the United States is primarily based on this effect. The similar impact is harder to establish in LMICs, as not all parents work full time and hence will not be absent from work.

Survey results showed that behavior-related productivity gains were considered to be least valuable for decision making. One possible explanation cited by an interviewee is that behavior-related productivity gains are very difficult to measure. There are also many social and cultural issues that can confound this relationship. For example: “In Brazil there is high vaccine coverage and high participation of women in the job market than next door to Bolivia with bit lower vaccine coverage and probably much lower participation in the job market. But then Bolivia’s economy is not so strong, culturally women’s have another position in society. Female education is lower in Bolivia, Types of work for [women] and kind of work is less. Is that because of vaccines or something else?” (sic).

#### Ecological effects

The impact of herd immunity on cost-effectiveness was seen as the most important ecological effect to measure. This is underlined by the fact that six respondents named it as a potential BEIV in the open questions. All three interviewees who discussed herd immunity thought it was an important ecological externality to measure in economic evaluations. Two cited the polio vaccine as an example where herd immunity is considered during the decision-making process in their country.

The effect of vaccination on reducing antimicrobial drug resistance was seen as the next most valuable outcome of an ecological effect. One survey respondent specified this effect in the open questions. Although, one interviewee reported that inclusion of the impact of a vaccine on drug resistance is only valuable in some cases, as in the malaria vaccine.

Serotype replacement was also deemed important albeit accompanied by a negative intonation with respect to vaccine advocacy. As one interviewee suggested, evidence demonstrating an eventual increase in detected cases of non-vaccine serotype cases (irrespective of occurrence of true replacement) would work against calls for increased vaccine provision and uptake.

#### Indirect effects

From the overall survey results, we found that indirect effects were one of the least important domains. Evidence of impact on equity, health resource utilization and priority of interventions were all scored with a median of three. Only evidence on the possibility of interaction with other interventions was considered important. However, in the open questions many respondents referred to different indirect effects. One interviewee stated that inaccurate calculation of costs and not accounting for hidden or unanticipated costs involved with vaccine implementation could be a potential issue.

The interplay between health rights and equity was also discussed by one interviewee, who stated “When implementing immunization programs it is very important to reach the lower socio-economic classes as the largest health gains can be obtained in this group”.

Positive externalities of vaccination programs in strengthening other health services were also mentioned during the interviews and in the open questions. Nine respondents indicated vaccines as an entry point or platform for providing a wide range of other public health interventions such as school-based and maternal health interventions. One interviewee gave some examples on how vaccine programs can improve health in other areas. “For example, when rotavirus vaccine was introduced in diarrheal disease surveillance and monitoring was improved. This had a positive effect on other disease surveillance in terms of training and education of staff in monitoring and evaluation etc.” Another interviewee also stressed that vaccines are normally not implemented as a single solution but are supported by other health interventions that target other causes of the disease. This possibility was also mentioned by three respondents of the survey.

Moreover, four interviewees saw the possibility of capacity building through education and training for health care workers. However, one interviewee warned against rolling out interventions without ensuring availability of trained personnel as this can create an additional burden on health care workers.

None of the interviewees or survey respondents thought investing in vaccines would result in a misdirection of focus, for example, using cholera vaccines as a quick and easy fix in place of investing in tackling a more fundamental health hazard such as clean drinking water and sanitation.

#### Macro-economic impact

Survey results showed that the potential burden stemming from preventable outbreaks of disease on other non-health sectors of the wider economy is highly relevant information. The education sector and the effect of school absenteeism was regarded to be important and could be one example of a non-health sector in a certain sense.

However none of the respondent mentioned economic impact on other sectors in the open questions and only one interviewee gave examples of the impact that vaccines may have on other sectors. Examples given were a hepatitis A virus and measles outbreak in an Eastern European country and an outbreak of polio virus, negatively affecting local fruit and vegetable markets.

Nonetheless, both survey respondents and interviewees reflected on the importance of evidence on school absenteeism of children. Two interviewees reported that the impact of vaccines on absenteeism offers a strong argument in favor of vaccine implementation, especially in the case of malaria. Furthermore, one interviewee testified towards a relationship between an NGO financed immunization program and reduced school absenteeism, eventually resulting in a positive impact on the overall economy of African countries. However, another interviewee did not agree with this point and doubted the validity of such a causal relationship.

### Other BEIVs

Interviewees mentioned some other possible positive effects of vaccination that are not usually discussed. One is the establishment of wide service network for children in the region. Another is the overall strengthening of local health care systems. Certain social goals can also be framed into becoming good rationales for vaccine uptake. For example, the HPV vaccine has been introduced in South America as it reduces mortality of young women of child-bearing age and hence the likelihood of orphaned children in the community.

One of the interviewees expressed concern that positive external are disproportionately measured in economic evaluation studies. This is a valid criticism considering, as one interviewee pointed out, that improper disposal of medical waste generated by the EPI program is a main issue in Africa. Sometimes used needles can be found on sites, which can potentially transfer pathogens from sick to healthy children.

### New tools for estimating BEIVs

None of the survey respondents or interviewees were able to propose new methods of evaluation. However, some interviewees and respondents advised on using some tools not included in the survey. These include PATH guidelines on economic evaluation of vaccines, the return on investment approach informing Ministry of Finance or Planning Departments on cost-effectiveness of vaccines and evaluating the effectiveness of vaccines in an individual country context during implementation. Furthermore, one interviewee mentioned vaccines should be evaluated in relation with social determinants of health. This idea is rooted in the belief that children do not die only from vaccine-preventable diseases but are also exposed to other risk factors.

### Area of core interest

Table 4 illustrates that in general, stakeholders working in LICs value evidence on burden of disease, ecological effects and macro-economic impact more highly than professionals working in MICs. For productivity-related gains, no differences could be found based on geographical working area. For the indirect effects, some differences could be identified between professionals working within a global context compared to professionals working in LICs, such as equity principles, which are more valued in LICs. However, only macro-economic impact is scored above four by the stakeholders working within a global context whilst representatives from MICs have scored ecological effects as a four.

**Table 4:** **Median scores per domain by geographical working area of core interest**

**Domains**	**Low income countries**		**Middle income countries**		**Global**	
	**N**	**Score**	**N**	**Score**	**N**	**Score**
Burden of disease	4	4.7	7	3.6	14	3.9
Productivity related gains	3	3.25	7	3.25	10	3.25
Ecological effects	3	5.00	7	4.00	13	3.66
Indirect effects	4	3.50	5	3.50	11	3.25
Macro-economic impact	4	4.50	7	3.50	12	4.25

### Different stakeholder groups

Table 5 gives an overview of the calculated importance of each domain specific to stakeholder group. Due to a lack of data on donors and manufacturers, scores for these stakeholders were not calculated. Respondents working in governmental bodies were seen to give higher scores to burden of disease, ecological effects and macro-economic impact. A similar trend was observed for respondents from international organizations and research institutes, although none of the domains were scored as very important for the latter group.

**Table 5:** **Median scores per domain by institutional background**

**Domains**	**Government**		**International organization**		**Research institute**	
	**N**	**Score**	**N**	**Score**	**N**	**Score**
Burden of disease	4	4.50	12	4.10	5	3.60
Productivity-related gains	4	3.88	9	3.25	5	3.00
Ecological effects	3	4.33	11	4.00	5	3.67
Indirect effects	4	3.25	10	3.38	4	3.25
Macro-economic impact	4	4.25	11	4.38	5	3.50

### Applicability to new versus traditional EPI vaccines

Four interviewees conveyed challenges with regards to the measurement of BEIVs for newer vaccines. Due to paucity in data, some interviewees recommended measuring BEIVs in older vaccines as the relevant economic and clinical data may be unavailable for new vaccines. There are also some inherent problems related to the type of diseases being targeted by newer vaccines. For example, benefits from the HPV and Hepatitis B Virus vaccines only occur much later in life. This increases the complexity of models involved and technical expertise required. In the case of rotavirus and pneumonia, reductions in mortality can only be observed at a global level as the disease is still widely prevalent at an individual or local level and utilizes many of the same resources (i.e health care workers, antibiotics). This factor affirms the relevance of including BEIVs in conveying the true value of new vaccines. Additionally, traditional EPI vaccines were also viewed as having a greater societal impact as they targeted diseases with greater incidence rates and which affected a greater proportion of children.

## Discussion

This study offers unique insight into the reasoning and justification employed by decision makers, funders and experts in the field of vaccine introduction.

According to respondents, the Ministries of Health, Finance and NITAGs play a crucial role in the current decision making process around new vaccine introductions. Although the numbers of NITAGs globally have been growing, the level of expertise in health economics has been limited and lacking in most LMICs [31]. Hence, explicit guidance on the inclusion of BEIVs by international organizations such as WHO would be most timely and well received.

Our study results indicate that evidence on burden of disease, safety and cost-effectiveness is regarded as most important for vaccine introduction decisions. This is in contrast to findings from another qualitative study which reported that only burden of disease evidence, and on occasion affordability and safety, was essential for immunization program decision making in seven LMICs [32]. More importantly, our study affirms that BEIVs belonging to ecological effects and macro-economic impact domains are considered to be equally as important as the more traditional outcome measures. Indirect effects and care-related productivity gains were considered less important to include in economic evaluation. Quite similarly, Burchett *et al*. [32] also reported that serotype replacement effects and, impact on non-health outcomes were rarely mentioned whereas equity considerations were only acknowledged in South Africa. Although productivity gains have often been cited in other studies [14,15,18,22,33], one explanation why this domain ranks lower on some studies [17] could be its relatively high importance to economists rather than clinicians and public sector officials who may be less familiar with its causal relationship. Cultural and contextual differences could be another possible explanation [4]. Indeed we observe that respondents dealing in an LIC context and representatives of government bodies tend to give higher importance to BEIVs than other stakeholders.

Considerations not covered by the survey were also brought up by interviewees. For example, the potential negative impact of wastage during immunization programs or the idea of using vaccines as a platform for providing other health interventions. The concept of combining vaccination with other interventions is not new [23]. However it remains difficult to translate the potential benefits of this synergistic effect into economic terms. Furthermore, interviewees highlighted the importance of using a broader perspective particularly when deriving the value of newer and costlier vaccines. This can be explained by the substantially higher list prices for new developed vaccines [34]. Several interviewees also reported the existence of different audiences. This finding underlines the necessity for flexible guidelines and the importance of combining different evaluation techniques to accommodate different needs and evaluation perspectives.

There are also several limitations to our study worth highlighting. Firstly, as most of the NUVI participants are actively involved in vaccine advocacy at a global as well as country level, the results may reflect biased viewpoints in favor of promoting the wider benefits of vaccines. Since this study was exploratory in nature, it was important to select a group that was actively engaged in decision making. It may be useful to conduct the survey within a more neutral group in the future. Secondly, the study suffered from a low response rate. Only 26 out of 140 respondents participated, which could be explained by the estimated 30 minute completion time. As a result none of the subgroup analyses performed were statistically significant. Future versions should attempt to be shorter. Offering rewards to attract participants could also be considered. Furthermore, the data collection could be organized in another setting. For example, the survey could be performed in workshop setting with direct feedback to the audience. Thirdly, the placement of the different types of impacts in the framework can be discussed. This is especially the case with school absenteeism which can also be placed under productivity-related gains and governmental savings which can be interpreted as a macro-economic impact. A separate analysis was performed to give insight in the outcomes for this alternative framework. It was found it would not change the results except for the macro-economic impact, which would be valued as even more important. Fourthly, using a Likert-scale has several methodological disadvantages [35]. These can be overcome by using a discrete choice experiment which would allow for a more explicit analysis of trade-offs between the various BEIVs and might offer greater discriminatory power than Likert-scales [36]. Finally, the qualitative part was supposed to add a layer on top of the survey findings, by providing illustrative anecdotal experiences and thoughts on feasibility of including BEIV in economic evaluation studies. However, it was difficult to conduct a sound comparison of the qualitative and quantitative part of the research, because not all interviewees filled out the survey. This may have resulted in recall bias as participants that contributed to both were already familiar with the different types of BEIVs outlined in the survey. To get more insight in this bias we checked for overrepresentation of both components in the results. No indications of this were to be found.

## Conclusion

Notwithstanding the limitations of this study, the uniqueness of this project should be considered as the study results will contribute to a better understanding by decision and policy makers regarding the usefulness of BEIVs information for vaccine introduction decisions at national level. Furthermore, these insights can also be used to shape upcoming research agendas in this field to facilitate creation of more comprehensive guidelines on economic evaluations of vaccines. However, several country-level studies should be conducted to test the proposed BEIVs on their ability to address the needs of different stakeholders and applicability to inform different audiences.

## Additional files

Additional file 1:
**Survey on the broader economic impact of vaccines and immunization programs.**
Additional file 2:
**Questions interviews.**

## Abbreviations

BEIV: Broader economic impact of vaccines
CBA: Cost benefit analysis
CEA: Cost effectiveness analysis
DALY: Disability adjusted life years
EPI: Expanded program on immunization
GAVI: Global alliance for vaccines and immunization
HICs: High income countries
HPV: Human papillomavirus
ICC: Inter-agency coordinating committees
LMICs: Low and middle income countries
LICs: Low income countries
MICs: Middle income countries
MSF: Medicines Sans Frontier
NGO: Non-governmental organization
NITAGs: National immunization technical advisory groups
NUVI: New and underutilized vaccine initiative
PAHO: Pan American Health Organization
QALY: Quality adjusted life years
UNICEF: United Nations International Children’s Emergency Fund
WHO: World Health Organization
