# Supplementary material for: Stakeholders’ perception on including broader economic impact of vaccines in economic evaluations in low and middle income countries: a mixed methods study
Source: BMC Public Health. 2015 Apr 10;15:356. doi: 10.1186/s12889-015-1638-0 (PMC4404665; doi:10.1186/s12889-015-1638-0)
Supplement: Additional file 1: — Survey on the broader economic impact of vaccines and immunization programs. [file 12889_2015_1638_MOESM1_ESM.pdf]

Survey on the broader economic impact of vaccines and immunization programs

Dear participant,

The WHO Immunization Vaccines and Biologicals Department in collaboration with Maastricht University, the University of Birmingham and the Health Protection Agency is conducting a study on the broader economic impact of vaccines and immunization programs. We would be very grateful if you could fill out this survey, which should take about 15 minutes of your time. Information provided by you will be treated confidentially and will be analyzed anonymously.

In middle and low income countries the resources to fund immunization programs are limited due to budget and other constraints. Currently, most economic evaluations of vaccination focus on their cost-effectiveness (i.e. how much it costs to obtain a certain amount of healthcare gain, such as a disability adjusted life year or DALY). In this study we want to investigate if broader economic considerations besides cost-effectiveness (e.g. budget impact, implementation issues, equity considerations) are important to decision makers and funders of immunization programs in low and middle income countries.

After completing the survey the printed version can be handed in by Ms Inge van der Putten during the NUVI meeting.

For questions please contact: *impactvaccines@who.int*

Part 1: In the first part of this questionnaire you will be asked some information about your personal background.

1. Gender

- □ Male
- □ Female

2. Age

…… years

3. How long have you been working[1] in the field of vaccination?

…… years

4. What is the name of the organization that you work for?
If you work for more than one organization, please choose the one which is most relevant to this survey.

………………………………………………………………………………………

5. What are your most important duties in the organization you work for?
Please provide a short job description.

………………………………………………………………………………………

6. In which of the following WHO region(s) do you work?
More than one answer is possible.

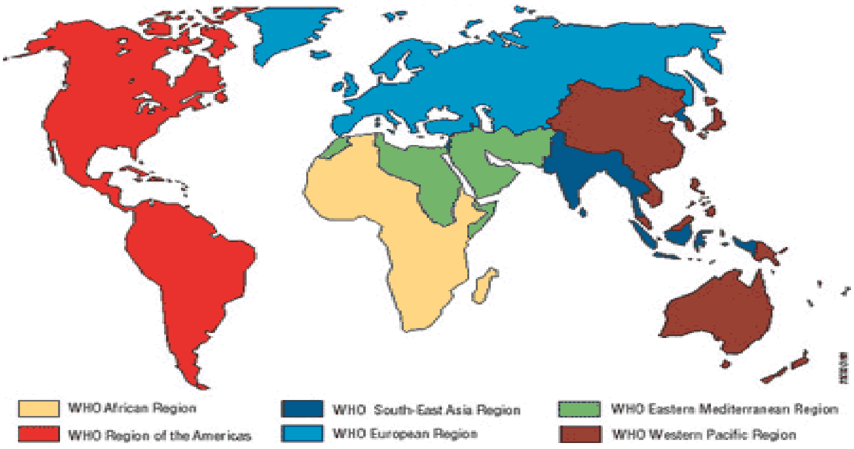

- □ WHO African Region
- □ WHO Region of the Americas
- □ WHO South-East Asia Region
- □ WHO European Region
- □ WHO Eastern Mediterranean Region
- □ WHO Western Pacific Region

[1] Excluding postgraduate research and/or medical residency (graduate medical training).

Part 2: In this part, you will be asked about the priority setting process in your country or the organization you represent in the country you work for.

7. How important are the following organizations in the decision making process for implementing immunization programs?
If you work in a high income country, please answer this question based on the low/middle income country most relevant to your work.
If any of the options are not applicable please circle "n/a".
If you are not familiar with any of the following organizations please circle "?".
The "others" option is to enable you to fill in any organizations not covered in options 1-6. If you cannot think of any more, please circle "n/a".

	**Not important**				**Very important**		
1. Parliament	1	2	3	4	5	n/a	?
2. Ministry of Health	1	2	3	4	5	n/a	?
3. Ministry of Finance	1	2	3	4	5	n/a	?
4. Special expert advisory group, namely ………………………………..	1	2	3	4	5	n/a	?
5. Non Governmental Organizations, namely …………………………………………	1	2	3	4	5	n/a	?
6. Local advocacy groups[2], namely …………………………......................	1	2	3	4	5	n/a	?
7. Others, namely …………………………………………	1	2	3	4	5	n/a	?
8. Others, namely ……………………………………........	1	2	3	4	5	n/a	?
9. Others, namely ……………………………………........	1	2	3	4	5	n/a	?

[2] e.g. patient groups.

8. How important are the following types of evidence when making decisions about immunization programs?
If you work in a high income country, please answer this question based on the low/middle income country most relevant to your work.
If any of the options are not applicable please circle "n/a".
If you are not familiar with any of the following types please circle "?".
The "others" option is to enable you to fill in any types not covered in options 1-6. If you cannot think of any more, please circle "n/a".

	**Not important**				**Very important**		
1. Effectiveness data[3]	1	2	3	4	5	n/a	?
2. Cost-effectiveness data[4]	1	2	3	4	5	n/a	?
3. Burden of disease[5]	1	2	3	4	5	n/a	?
4. Overall costs of an immunization program	1	2	3	4	5	n/a	?
5. Public sector budget impact	1	2	3	4	5	n/a	?
6. Accessibility[6]	1	2	3	4	5	n/a	?
7. Safety[7]	1	2	3	4	5	n/a	?
8. Applicability[8]	1	2	3	4	5	n/a	?
9. Credibility[9]	1	2	3	4	5	n/a	?
10. Equity, fairness[10]	1	2	3	4	5	n/a	?
11. Availability of other interventions[11]	1	2	3	4	5	n/a	?
12. Others, namely …………………………………….......	1	2	3	4	5	n/a	?
13. Others, namely …………………………………………	1	2	3	4	5	n/a	?
14. Others, namely …………………………………………	1	2	3	4	5	n/a	?

[3] Vaccine effectiveness as reported in clinical trials, observational studies and post-marketing surveillance.
[4] Results from studies comparing the cost of an immunization program with its benefits (measured in units such cases avoided, deaths prevented or DALYs gained).
[5] Estimates of total morbidity and mortality due to the disease.
[6] Availability of the vaccine, taking into account social, cultural, economic and geographical barriers to being vaccinated.
[7] Reported adverse effects of the vaccine.
[8] Whether the evidence presented was generated in a setting comparable to that in your own country (in terms of disease epidemiology, economic status and cultural norms/values).
[9] Whether the authors and/or institutions generating or publishing the evidence are regarded as objective, unbiased and respected.
[10] The extent to which an immunization program will improve the health of everyone equally.
[11] Whether or not other interventions exist besides vaccination that may reduce the incidence and/or severity of the disease.

Part 3: This part involves questions about the effects of an immunization program other than the direct health benefits to the people being vaccinated. Such effects can be either beneficial or harmful.

9. Are there any effects of an immunization program that you consider to be important, other than the direct health benefits to the people being vaccinated?
Please make a list of the effects from most important to least important

……………………………………………………………………………………………

……………………………………………………………………………………………

……………………………………………………………………………………………

……………………………………………………………………………………………

……………………………………………………………………………………………

……………………………………………………………………………………………

……………………………………………………………………………………………

……………………………………………………………………………………………

……………………………………………………………………………………………

……………………………………………………………………………………………

……………………………………………………………………………………………

……………………………………………………………………………………………

……………………………………………………………………………………………

……………………………………………………………………………………………

……………………………………………………………………………………………

10. Suppose the following statements about burden of disease are made about the impact an immunization progra being considered in your country. In your opinion, how important is each piece of information to such a decision?
If you work in a high income country, please answer this question based on the low/middle income country most relevant to your work.
If any of the options are not applicable please circle "n/a".
If you are not familiar with any of the following scenarios please circle "?".

**Scenarios**	**Not important**				**Very important**		
1. An immunization program for vaccine X is estimated to reduce the number of people who contract the relevant disease by 20%.	1	2	3	4	5	n/a	?
2. An immunization program for vaccine X is estimated to reduce the number of deaths due to the relevant disease by 5%.	1	2	3	4	5	n/a	?
3. An immunization program for vaccine X is estimated to increase the health-related quality of life of the population by 10%.	1	2	3	4	5	n/a	?
4. An immunization program for vaccine X implemented is estimated to reduce total healthcare costs for the relevant disease by 5%.	1	2	3	4	5	n/a	?
5. Implementation of an immunization program for vaccine X is expected to bring about government savings (across all departments) of $2 million a year.	1	2	3	4	5	n/a	?

11. Suppose the following statements are made about the productivity-related gains of a vaccine which is being considered in your country. In your opinion, how important is each piece of information to such a decision?
If you work in a high income country, please answer this question based on the low/middle income country most relevant to your work.
If any of the options are not applicable please circle "n/a".
If you are not familiar with any of the following scenarios please circle "?".

Scenarios	Not important				Very important		
1. When an immunization program for vaccine X is implemented, the average parent or caregiver may save 6 hours a year by avoiding the need to take care of a sick child.	1	2	3	4	5	n/a	?
2.The implementing of an immunization program for vaccine X may increase the lifetime labour productivity of vaccinated children because vaccination will improve their cognition and physical strength, as well as school enrolment, attendance and attainment.	1	2	3	4	5	n/a	?
3. The implementing of an immunization program for vaccine X may eventually influence choices on fertility and hence improve participation of women in the labour market.	1	2	3	4	5	n/a	?
4. The implementing of an immunization program for vaccine X may influence choices on fertility and hence improve the nutrition, health and educational attainment of children. This can in turn increase the child's labour productivity as an adult.	1	2	3	4	5	n/a	?

12. Suppose the following statements are made about the ecological effects of a vaccine which is being considered in your country. In your opinion, how important is each piece of information to such a decision?
If you work in a high income country, please answer this question based on the low/middle income country most relevant to your work.
If any of the options are not applicable please circle "n/a".
If you are not familiar with any of the following scenarios please circle "?".

**Scenarios**	**Not important**				**Very important**		
1. Implementing an immunization program for vaccine X may reduce/delay the emergence of drug resistant strains in the region.	1	2	3	4	5	n/a	?
2. Implementing an immunization program for vaccine X may lead serotype replacement effects in the population i.e. an increase in disease caused by serotypes not covered by the vaccine.	1	2	3	4	5	n/a	?
3. Implementing an immunization program for vaccine X may reduce the risk that non-vaccinated individuals will acquire the disease.	1	2	3	4	5	n/a	?

13. Suppose the following statements are made about the indirect effects of a vaccine which is being considered in your country. In your opinion, how important is each piece of information to such a decision?
If you work in a high income country, please answer this question based on the low/middle income country most relevant to your work.
If any of the options are not applicable please circle "n/a".
If you are not familiar with any of the following scenarios please circle "?".

Scenarios	Not important			Very important			
1. Implementing an uptake for vaccine X may be highest in protecting people with lowest risk of disease (due to their socioeconomic and educational advantage over non-vaccinated people).	1	2	3	4	5	n/a	?
2. Other preventative or therapeutic interventions for the same disease may be introduced, which would reduce the benefit of an immunization program for vaccine X.	1	2	3	4	5	n/a	?
3. Implementing an immunization program for vaccine X would reduce the time that healthcare workers have.	1	2	3	4	5	n/a	?
4. Implementing an immunization program for vaccine X may reduce the priority given to wider public health interventions to reduce disease incidence (such as sanitation, maternal health improvement and provision of clean water).	1	2	3	4	5	n/a	?

14. Suppose the following statements are made about the impact of a vaccine on other sectors, which is being considered in your country. In your opinion, how important is each piece of information to such a decision?
If you work in a high income country, please answer this question based on the low/middle income country most relevant to your work.
If any of the options are not applicable please circle "n/a".
If you are not familiar with any of the following scenarios please circle "?".

Scenarios	Not important				Very important		
1. Implementing an immunization program for vaccine X may reduce the financial burden on other sectors besides healthcare.	1	2	3	4	5	n/a	?
2. Implementing an immunization program for vaccine X may reduce school absenteeism.	1	2	3	4	5	n/a	?

15. Do you know of any information about the broader impact of vaccines and / or immunization programs in your country?

- ◻ No
- ◻ Yes, namely

………………………………………………………………………………………

………………………………………………………………………………………

………………………………………………………………………………………

………………………………………………………………………………………

………………………………………………………………………………………......

....

....

....

16. Can you think of any original methods to measure the broader impact of vaccines and / or immunization programs?

- ◻ No
- ◻ Yes, namely

………………………………………………………………………………………

………………………………………………………………………………………

………………………………………………………………………………………

………………………………………………………………………………………

………………………………………………………………………………………

………………………………………………………………………………………

………………………………………………………………………………………

………………………………………………………………………………………

17. Are there any other examples of the broader impact of vaccines and/or immunization programs that have not been mentioned in questions 9-14?

- ◻ No
- ◻ Yes, namely

………………………………………………………………………………………

………………………………………………………………………………………

………………………………………………………………………………………

………………………………………………………………………………………

………………………………………………………………………………………

………………………………………………………………………………………

………………………………………………………………………………………

………………………………………………………………………………………

It would be very helpful if we could interview you about your thoughts on this subject, to obtain greater insight about the considerations underlying your answers to this questionnaire, and how different kinds of vaccines may merit different considerations.

Interviews will be conducted during the NUVI meeting in Montreux on 22-23 June, and will take about 20 minutes. If you are happy to be interviewed, please fill out the contact information below:

18. Name

…………………………………………

19. E-mail address

…………………………………………

If you like to be informed about the results of this survey, please leave your e-mail address so that we can contact you.

20. E-mail address

…………………………………………

Thank you for the time taken to fill out this questionnaire. The information provided will help the WHO to understand key considerations in vaccine implementation, and to develop its work program around these issues. The printed version of the survey can be handed in by Ms Inge van der Putten during the NUVI meeting.

If you still have any questions or comments on the research please contact; impactvaccines@who.int
