# Supplementary material for: Stakeholders’ perception on including broader economic impact of vaccines in economic evaluations in low and middle income countries: a mixed methods study
Source: BMC Public Health. 2015 Apr 10;15:356. doi: 10.1186/s12889-015-1638-0 (PMC4404665; doi:10.1186/s12889-015-1638-0)
Supplement: Additional file 2: — Questions interviews. [file 12889_2015_1638_MOESM2_ESM.pdf]

Questions interviews

Introduction:

Master students Maastricht University, Global health Health, Policy and Innovation Management.
Maastricht University works on this project in collaboration with WHO Immunization Vaccines and Biologicals department in University of Birmingham and the Health Protection Agency

Need:
In middle and low income countries the resources to fund immunization programs are limited due to budget and other constraints. Currently, most economic evaluations of vaccinations focus on their cost-effectiveness. In this study we want to investigate if any other considerations besides cost-effectiveness (e.g. budget impact, implementation issues, equity considerations) are important to decision makers and funders of immunization programs in low and middle income countries.

Timeframe interview: 15 min
Is it okay to record the interview?

Interview (start with 1 if more time is available, ask next question):

1. Additional information on external effects (question 10-14 & did we miss some types of effects)
    a. Do you want to further elaborate on a few things you filled out in the survey and that you think need some more explanation?
    b. Did you miss a type of effect in the survey that you came across in the years you worked in the field of vaccines?
2. Any considerations on methods to measure effects for in the future
    a. Can you give us some recommendations on how to measure some sort of external effects in the future?
    b. Is there as far as you know any data/literature available that we can use for further research
3. Differences between vaccines (traditional vs new developed)
    a. Are there different issues important for different vaccines?
